# A Mixed Methods Evaluation of Ecological Momentary Assessments: The Impact of Self-Monitoring and Lifestyle Education in Patients With Uncontrolled Hypertension

**DOI:** 10.7759/cureus.85734

**Published:** 2025-06-10

**Authors:** Sonal J Patil, Ning Guo, Irina Todorov, Douglas Gunzler

**Affiliations:** 1 Family Medicine, MetroHealth System and Case Western Reserve University School of Medicine, Cleveland, USA; 2 Quantitative Health Sciences, Cleveland Clinic, Cleveland, USA; 3 Integrative Medicine and Health, Mayo Clinic, Jacksonville, USA; 4 Epidemiology and Public Health, Center for Health Care Research and Policy, Metrohealth Medical Center, Cleveland, USA

**Keywords:** ecological momentary assessments, hypertension, mixed-methods, primary care, self-monitoring

## Abstract

Background: Psychological stress is known to influence blood pressure (BP) and cardiovascular health, yet limited research has explored real-time associations between emotions, lifestyle behaviors (diet and physical activity), and BP in diverse primary care populations with uncontrolled hypertension. This study evaluated the relationship between momentary emotions, diet, physical activity, and home BPs in patients participating in a six-week self-monitoring program that included ecological momentary assessments (EMAs) and coping skills and lifestyle education.

Methods: Patients with uncontrolled hypertension (defined as BP>130/80 mm Hg) who preferred lifestyle changes over medication intensification participated in a feasibility study involving EMA-based self-monitoring of BP, diet, physical activity, sleep, and emotions. Linear mixed effects models and time-varying effect models (TVEMs) were used to examine change over time in negative affect, positive affect, diet, physical activity, sleep, and home BPs. A convergent mixed methods design was used, combining linear mixed effects models to analyze BP trends and thematic analysis of qualitative participant feedback.

Results: A total of 29 participants (mean age: 55.3±10.6 years, 22 (75.9%) female, 13 (44.8%) Black) completed the intervention. Systolic and diastolic BP and positive affect changed significantly over time (p<0.0001). No significant changes were observed in diet, sleep, physical activity, and negative affect within day or in-between days over time. TVEMs showed that increasing positive affect was associated with lower home BPs and increasing negative affect was associated with higher home BPs. Qualitative analysis revealed that most participants perceived negative emotions influenced BP and health behavior choices. However, some participants who maintained consistent health behaviors reported minimal emotional effects on BP.

Conclusions: In diverse primary care patients with uncontrolled hypertension, momentary home BP and positive emotions improved over time with the self-monitoring and education intervention, while negative emotions remained unchanged. Patient-perceived emotions affected health behaviors and BP. Healthy habits were perceived to attenuate the effect of emotions on BP. Future studies should consider a longer duration of intervention and additional instrumental support for negative affect and lifestyle behavior modifications to jointly improve cardiovascular and psychological health. Larger future studies are needed to better understand the direct and indirect relationships between emotions, lifestyle behaviors, and home BPs.

## Introduction

Psychological stress triggers physiological responses, which are postulated to increase cardiovascular disease risks and hypertension [1]. Negative emotions adversely impact cardiovascular health through behavioral, physiologic, and neurobiological mechanisms. Emotional reactivity of blood pressure (BP) has been widely studied and intervened on using ecological momentary assessments (EMAs) [2]. However, most previous EMA studies have been in White and non-clinical populations or populations with mental health and pain-related chronic conditions, did not include monitoring of cardiovascular health behaviors, and did not target lifestyle education to improve hypertension [3-5]. Studies targeting stress reduction techniques showed improved clinic-based BP, but out-of-office BPs, which are known to be more accurate, were rarely measured [6].

To address these gaps, we explore the associations between emotions and diet, physical activity, and home BPs in patients with uncontrolled hypertension participating in a six-week program of self-monitoring of BPs, behaviors, and emotions combined with coping skills and lifestyle education. The primary research question for our study is to explore how direct and indirect relationships between emotions, diet, physical activity, and home BPs evolve over time during participation in a six-week self-monitoring and education program in patients with uncontrolled hypertension. Our secondary aim was to explore the feasibility of EMA data collection and analysis using a time-varying effects model to demonstrate proof-of-concept for improvements in emotions, lifestyle behaviors, and BPs over the course of a self-monitoring and education program.

## Materials and methods

Our study includes patients with uncontrolled hypertension who preferred lifestyle changes over medication intensification and participated in a feasibility study of a self-monitoring program that integrates education on coping strategies and lifestyle modifications. A control group was not feasible as the participation was based on patient preference for lifestyle modification over medication intensification. The program was designed using the self-regulation model from Bandura’s social cognitive theory of an agentic perspective, which focuses on continuous interactions between individuals, the environment, and behaviors. A detailed description of the intervention is provided in the previously published results of the feasibility and acceptability of our self-monitoring and education program [7]. In brief, patients with uncontrolled hypertension and interested in lifestyle modifications instead of medications were enrolled and taught to self-report home BPs, sleep, diet, physical activity behaviors, and emotions using EMAs. The six-week program included weekly resources such as healthy recipes, links to cooking videos, audio and video guided imagery, and yoga videos. The education promoted awareness of how emotions can influence health-related behaviors, with a focus on four key components of wellbeing: nutrition, physical activity, stress relief, and restorative sleep.

A validated home BP monitor was loaned to all patients for six weeks. We used the American Heart Association’s (AHA) cardiovascular health components for lifestyle modifications, physical activity as the number of minutes of moderate or vigorous physical activity, and diet as combined components of serving sizes of vegetables, fruits, whole grains, fish, and soda intake [8]. Emotions were monitored using the positive and negative affect schedule (PANAS), a validated self-report scale for emotion monitoring. We used the REDCap platform, MyCap, which allows the development of online assessments to be completed remotely by participants by email or through push notifications on an app [9]. MyCap is an iPhone® and Android application that can be downloaded from the Apple Store® and Google Play®. MyCap sent push notifications to participants daily with a morning, afternoon, and evening survey. Patients completed self-reports of emotions, health behaviors, and BP while receiving education on coping skills and lifestyle modifications for six weeks. The survey platform setting required participants to complete all the questions before submitting or canceling survey completion; hence, we did not have any incomplete survey responses.

We have a detailed Modified CONSORT (Consolidated Standards of Reporting Trials) flow diagram showing screening, enrollment, follow-up, and analysis of participants in the previously published results [7]. In summary, over six months, 31 participants completed the program (mean age 56 years (SD 11.1), 23 (74.2%) women, 13 (41.9%) Black adults). After completing the six-week intervention, 28 participants completed an open-ended online post-intervention survey (for details, see the supporting information of [7]), and one participant provided feedback through an interview. Post-intervention questions asked participants their opinions on how their mood affects their diet, physical activity, and BP; how their diet affects their BP; and how their physical activity affects their BP.

Analysis

We used a convergent mixed methods design where qualitative and quantitative data were merged to provide a better understanding of how emotions and behaviors affect home BPs.

Quantitative Data Analysis

This was an exploratory evaluation of a feasibility study, so the sample size was based on the goal of evaluating the feasibility of self-monitoring using EMA in patients with uncontrolled hypertension and available funding resources. Most EMA studies have sample sizes <100 with a median sample size of 40 in EMA studies with clinical participants, and we had 29 eligible participants who completed a median of 87 EMAs over four to six weeks [10,11].

Data Cleaning and Exclusion

Data from MyCap was collected with time stamps with data reported before 12 noon as morning data, data reported between 12 pm and 4 pm as afternoon data, and data after 4 pm labeled as evening data. Data was cleaned and stacked as morning, afternoon, and evening EMA data. As we were interested in studying within-person and between-person associations and changes over time with our intervention, we analyzed individuals with more than four days of self-monitoring data. We used a total of 2321 observations from 29 participants, with the median number of observations per participant being 87[IQR 50 to 112]. All analyses were conducted using SAS.

Data Analysis

We report the descriptive information of the included adults.

Preliminary Trend Analysis

Preliminary analysis was done to determine which outcomes changed over time. We first examined whether there is significant variation or change over time in negative affect, positive affect, diet, physical activity, sleep, and home BPs. We ran linear mixed effects models. The linear mixed model contains fixed effects that represent mean responses, whereas the random effect represents the within-person responses. We calculated an intraclass correlation coefficient (ICC) to evaluate within-subject variation over the day for the EMA data. We did not find significant changes in diet, sleep, physical activity, and negative affect within day or in-between days over time. There was improvement noted in home BP and positive affect over time of our intervention duration.

Primary Analysis

We looked at baseline correlations among those that didn’t change over time (negative affect, diet, and physical activity) and the time-varying effect model for those that did (positive affect and BP). Spearman’s correlation was used to assess relationships between baseline negative affect, diet, and physical activity with BP. Time-varying effect models (TVEMs) were used to examine the time-varying effects of positive affect on home BPs over intervention participation. Finally, we conducted exploratory mediation analysis to evaluate the potential mechanisms of diet and physical activity’s impact on change in BP via negative and positive affect.

Qualitative Data Analysis

The survey qualitative data and the single interview data were extracted in Microsoft Word (Microsoft Corporation, Redmond, USA). We initially reviewed the data in-depth and coded manually as we had a manageable data size and analyzed each participant as a case, reflecting on their data across survey questions. We coded participant experiences of the impact of emotions on diet, physical activity, and home BPs. Thematic analysis was used to identify key themes related to our research questions. Two researchers with expertise in hypertension and integrative medicine read the transcripts and coded the data independently, followed by collapsing the codes into potential themes and subthemes. Codes and themes were reviewed and refined consecutively by discussion between the two coders to gain inter-coder consensus. We assessed themes guided by our main question of how participants perceive their emotions as impacting diet, physical activity, and home BPs.

## Results

Twenty-nine participants (22 [75.9%] female, 13 [44.8%] Black, mean age 55.3±10.6) who had completed EMA and provided post-intervention insights from program participation were included in the analysis (Table 1). Of the 29 participants, 25 (86.2%) completed at least three days of EMA per week for four weeks.

**Table 1:** Descriptive statistics for the sample

Descriptive statistics for the sample	Mean/N		SD/%		Range
Age					
< 50 years, N(%)	9		31.0		(38,47)
>=50 years, N(%)	20		69.0		(50,72)
Gender					
Male (%)	6		20.7		
Female (%)	22		75.9		
Other	1		3.5		
Race/Ethnicity					
White (%)	16		55.2		
African-American/Black (%)	13		44.8		
Education					
High school graduate, diploma, or the equivalent (GED)	3		10.3		
Some college credit (no degree)	3		10.3		
Associate degree	2		6.9		
Bachelor's degree	10		34.5		
Master's degree	10		34.5		
Missing	1		3.5		
Marital status					
Married	15		51.7		
Single/Widowed/Divorced	14		48.3		

Multilevel models

Table 2 shows EMA level descriptive statistics.

**Table 2:** EMA level descriptive statistics EMA: Ecological momentary assessment

EMA levels	Mean	SD
Systolic BP	132.3	12.2
Diastolic BP	84.3	10.1
Positive affect	21.2	10.2
Negative affect	11.6	3.4
Diet (Number of heart healthy components)	1.6	1.0
Physical activity (number of minutes	66.8	108.9

Linear mixed effects models showed that systolic BP (p < 0.0001) and diastolic BP (p<0.0001) changed significantly with time. Negative affect did not change over time (p=0.83), whereas positive affect did change over time (p<0.0001). The ICCs suggest that 41.1% of the total variance in systolic BP was within participants and 58.9% variance was between participants. Similarly, 27.5% and 72.5% variance for diastolic BP was within and in-between participants, and 35.3% and 64.7% variance for positive affect was within and in-between participants. Systolic BP, diastolic BP, and positive affect improved over the intervention period. We checked for correlations between average negative affect, diet, and physical activity with BP using Spearman’s correlation and did not find any significant correlations as these variables did not change with time (Table 3).

**Table 3:** Association between negative affect, diet, physical activity and systolic and diastolic BP BP: Blood pressure

Variables	Systolic BP		Diastolic BP	
	Spearman correlation	p-value	Spearman correlation	p-value
Negative Affect	0.16	0.40	0.12	0.57
Diet	0.15	0.43	0.16	0.40
Physical activity	-0.19	0.31	0.12	0.54

TVEMs showed decreasing BP with improving positive affect over the intervention duration, whereas increased negative affect was associated with higher BPs (Table 4).

**Table 4:** Time-varying effects over the intervention duration ICC: Intraclass correlation coefficient

EMA levels	Estimate	Standard Error	p-value	ICC (patient level)
Systolic BP	-0.08566	0.01649	<0.0001	0.58852
Diastolic BP	-0.05166	0.01054	<0.0001	0.724672
Positive affect	0.06975	0.01149	<0.0001	0.646682
Negative affect	0.001042	0.004887	0.83	0.441901
Diet (number of heart healthy components)	-0.00276	0.002115	0.19	0.459051
Physical activity (number of minutes	0.07885	0.09130	0.39	0.240595

Exploratory mediation analysis, examining negative and positive affect as potential mediators between diet and physical activity levels and BP, did not yield statistically significant results.

Qualitative analysis

We identified themes indicating the presence or absence of the effect of emotions on diet, physical activity, and BPs. Negative emotions were frequently associated with blood pressure and health behaviors, while positive emotions were mentioned less often by study participants. Themes revealed three emotion-BP relationship patterns: indirect (through the effect of emotions on behaviors), direct (emotions affecting BPs), or effects of emotions attenuated by healthy habits.

Theme 1

Theme 1 details indirect relationships, i.e. interplay of emotions with diet and physical activity, which in turn may affect BPs.

Many patients considered emotions to affect their BP by interacting with their diet and physical activity choices.

Participant 2: “It (emotion monitoring) showed me why I am eating the way I did. The kind of snacks I was reaching for….If my eating habits are not right, this will impact my BP… emotions. can lead to bad habits all around.”

Participant 16: “When I'm stressed it has a negative effect (on BP), when I'm in a good mood it has a positive effect. It's all tied together. When I'm in a good mood, I eat good and am willing to do physical activity. When I'm not in a good mood, I tend to eat things I know I shouldn't and don't want to do anything physical…When I eat certain foods, it affects my blood pressure negatively. When I walk, it helps my blood pressure.”

*Theme 2* 

Theme 2 details direct relationships, i.e., emotions directly affect BPs.

Several patients perceived direct effects of negative and positive emotions on BPs.

Participant 26: “I noticed that mood affected my blood pressure, on the afternoon, if I was frustrated or aggravated, it was up, so I would wait and calm down and then retake… Didn't really notice diet affecting blood pressure at all, maybe affects it a little bit. Stress level and mood affect it the most.”

Participant 32: “made me more aware of my mood and positive or negative interpersonal interactions and how it made me feel and changed my BP.”

Theme 3

Theme 3 details effects of emotions attenuated by healthy habits, i.e., emotions may not affect BP if following healthy diet and physical activity behaviors.

Patients who did not perceive the effect of emotions on BPs typically focused on healthy diet and physical activity levels.

Participant 11: “not sure (effect of emotions on BP). I am pretty consistent with exercise and relatively disciplined on diet. Salt/ Sodium/eating at a restaurant appear to have a major impact. Guess that my average top number is around 130. Last night, when I got back from soccer it was 107 (had already logged my night). I am typically lower post exercise is the point.”

Participant 22: “my mood didn’t really change much so did not notice much change… noticed that salt is a factor. know that with physical activity my blood pressure would get high...goes high right after but then goes down.”

Convergent mixed methods analysis

TVEMs showed that higher positive affect was associated with lower BPs and higher negative affect was associated with higher BPs. Qualitative themes revealed direct and indirect emotion-BP relationship patterns but patients maintaining healthy lifestyles did not perceive emotional effects on BP.

**Figure 1:**
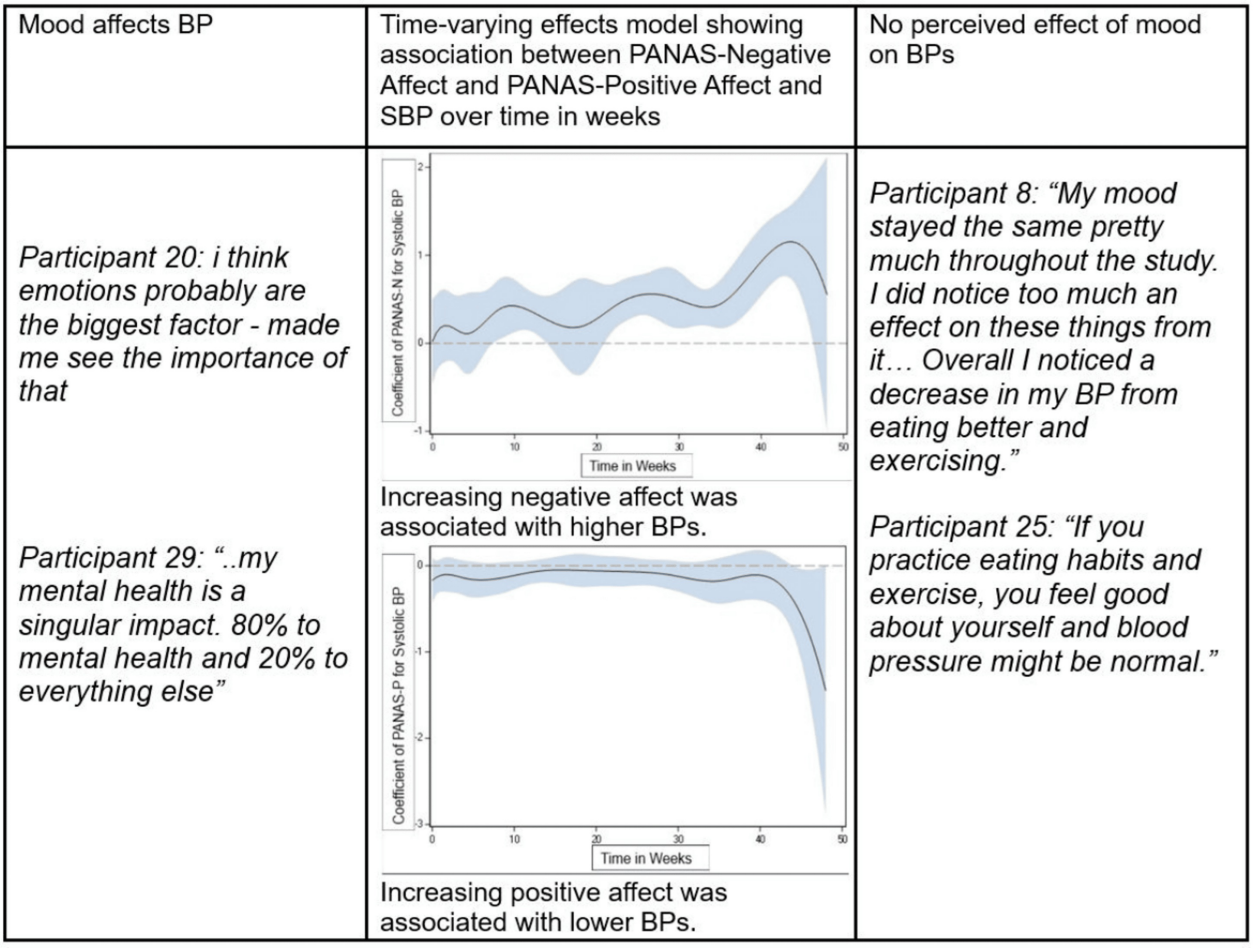
Joint display of overall quantitative and qualitative findings

Figure 1 shows the joint display of overall quantitative and qualitative findings with TVEM graph for change in PA and NA and systolic BP.

## Discussion

Our study is the first to look at momentary associations between emotions and BPs in patients with uncontrolled hypertension when participating in education on coping skills and lifestyle modifications. Our results showed that momentary positive emotions and BPs improved in diverse primary care patients with uncontrolled hypertension with a self-monitoring and lifestyle modification intervention. While there is significant observational research on the effect of positive emotions and cardiovascular disease risks, studies rarely combined interventions targeting health behaviors and psychological well-being to demonstrate improvement in cardiovascular risks and positive emotions [12-15]. To our knowledge, this is the first EMA study to show self-monitoring and education improved BPs and patient experiences of positive emotions in a diverse primary care patient population with uncontrolled hypertension. Most patients perceived emotions as influential on their lifestyle and blood pressure adding participant voice to our quantitative findings.

While quantitative measures showed a trend of association and improving positive affect and BPs over time in our study, negative affect, diet, and physical activity showed limited variability across time, and no significant associations with BPs emerged. Previous research reported reduced physiological reactivity to negative emotions and improved positive emotions in older adults; our study mainly included individuals above 50 years of age [16]. Reduced cardiovascular adaptability due to dysregulated autonomic nervous systems is known to be associated with hypertension and consistent with our finding of lack of correlation between negative affect and BPs in patients with uncontrolled hypertension, though our small sample size could have precluded these findings [17].

We did not find evidence of significant mediation effects of negative or positive affect in the relationship between diet and physical activity on BP, but given the exploratory nature of this analysis and small sample size, these non-significant findings should be interpreted with caution. A larger sample size, specific measures of affect, and more targeted interventions could help clarify the role of affect in mediating the relationship between behavioral changes and BP regulation.

Our qualitative evaluation revealed that patients frequently perceived emotional experiences directly influenced their BP or impacted their decisions regarding diet and physical activity, which subsequently affected their BP. Participants who self-perceived following a healthy diet and regular physical activity reported minimal impact of emotions on their BP with mostly stable emotions. In line with our findings, a recent systematic review and network meta-analysis demonstrated that the effects of exercise are comparable to pharmacotherapy or psychotherapy for treating depression [18]. Future studies should focus on modifying health behaviors to improve psychological and cardiovascular health.

Our quantitative evaluation showed an improvement in positive affect with more between-person variability, and more than half of the variability in negative affect was due to within-person fluctuations over time. Qualitatively, patients who perceived emotions as influencing their BP frequently focused on negative emotions. Our intervention may have heightened patients’ self-awareness of positive emotions, contributing to improved BP, but without significantly altering negative emotions. Previous studies have similarly found that positive emotions can mitigate the effects of depression on overall well-being and improve health behaviors [19,20].

Strengths

This is the first study to use the TVEM to study the effects of emotions and home BPs over the course of self-monitoring and education program participation. We add to the limited (nonexistent) literature of EMA studies in patients with uncontrolled hypertension, with most participants being over 50 years of age (n=20, 69.0%). Previous EMA research has largely involved healthy individuals or relied on self-reported hypertension [4,5]. We are the first EMA study to explore psychological and cardiovascular health and well-being in diverse primary care clinical populations with uncontrolled hypertension. We used a mixed methods approach to explain associations and add participant voice to our EMA analysis, providing a rich description of how emotions may affect diet, physical activity, and BPs.

Limitations and future research directions

The changes in diet, physical activity, and negative affect may not have been significant over time due to the short duration of the intervention and lack of instrumental support for behavior modifications. Future studies should explore the addition of instrumental support, such as medically tailored groceries, links to physical activity programs, or wearable sensors that provide real-time feedback on physiologic responses to emotions and monitor physical activity levels. Moreover, digital mental health apps and just-in-time interventions to support the management of negative emotions beyond self-monitoring and education may need to be explored. Lastly, psychological literature suggests that meaningful improvement in conditions like depression typically requires at least 8-12 weeks of consistent psychotherapy. However, we chose a six-week intervention because shared medical appointment programs showed decreased attendance and engagement after six weeks.

We may not have noted significant associations between negative emotions, diet, and physical activity with BPs due to the small sample and reduced variability in cardiovascular reactivity in patients with uncontrolled hypertension who are typically older adults. However, while PANAS is a well-validated scale, it was not perceived as patient-centered for multiple daily assessments of emotions [7]. Using brief patient-centered emotion scales for EMA may improve practicality and assessments of the effects of emotions on cardiovascular health behaviors. Further studies with larger samples and extended durations are needed to explore how cardiovascular health behaviors evolve over time with intervention and how these behaviors relate to emotional experiences and psychological well-being. 

## Conclusions

Momentary positive emotions and home BPs improved in diverse primary care patients with uncontrolled hypertension who participated in self-monitoring with coping skills and lifestyle education intervention. Interventions that promote real-time self-awareness of emotions, behavior, and BP, combined with education, may improve psychological well-being and hypertension outcomes in primary care settings. Future larger controlled studies are needed to identify how direct and indirect relationships between emotions, diet, physical activity, and home BPs evolve over time with self-monitoring and education interventions. Future studies should consider a longer intervention duration and additional instrumental support to improve negative affect and health behavior outcomes. Participants who maintained a healthy diet and engaged in regular physical activity reported experiencing fewer emotional impacts on their cardiovascular health. Future studies need to consider lifestyle modification interventions to improve mental and cardiovascular health.
